# Technology in Care Access and Use Among Adults With Cognitive Limitations During the Pandemic – Scoping Review

**DOI:** 10.1093/geroni/igaf122.1349

**Published:** 2025-12-31

**Authors:** HwaJung Choi, Mohsen Najar, Rahat Naseem, Hisham Irshaid, Tina Mainieri

**Affiliations:** University of Michigan, Ann Arbor, Michigan, United States; University of Michigan, Ann Arbor, Michigan, United States; University of Michigan, Ann Arbor, Michigan, United States; University of Michigan, Ann Arbor, Michigan, United States; University of Michigan, Ann Arbor, Michigan, United States

## Abstract

Many care services and social programs were halted or reduced during the COVID-19 pandemic. Online platforms for care delivery and social connections were introduced. It is important to understand the experience with the new technology by those with cognitive limitations in accessing care and support services. We reviewed 68 published articles that examined and discussed technology in the context of care availability and utilization among adults with cognitive limitations during the COVID-19 pandemic. There was overwhelming evidence of increased exposure to technology in care services and social programs – physician consultation, community programs, and contact with family and friends. Some suggest that remote care services played a significant role in filling the gap, such as prescriptions, during the pandemic. However, about 30% of the articles mentioned limitations in the use of technology. Adapting to a remote care system was challenging for adults living with cognitive impairments, including cognitive fatigue and difficulties with navigation. Additional health problems, in particular hearing and vision impairments, exacerbated the challenge with online consultation and social engagements. Family caregivers also experienced limitations and challenges due to their lack of familiarity with technology. The use of some services (e.g., telemedicine) declined as the state of emergency was lifted. A prompt shift to remote care delivery system, assisted with new technology, was necessary during the pandemic. While it played a substantial role in filling the gap in care services and social programs, careful assessments of the effectiveness of remote systems are needed specifically for those with cognitive limitations.

